# Biological function integrated prediction of severe radiographic progression in rheumatoid arthritis: a nested case control study

**DOI:** 10.1186/s13075-017-1414-x

**Published:** 2017-10-25

**Authors:** Young Bin Joo, Yul Kim, Youngho Park, Kwangwoo Kim, Jeong Ah Ryu, Seunghun Lee, So-Young Bang, Hye-Soon Lee, Gwan-Su Yi, Sang-Cheol Bae

**Affiliations:** 1 Department of Rheumatology, St. Vincent’s Hospital, The Catholic University of Korea, Suwon, Republic of Korea; 2 Department of Bio and Brain Engineering, Korea Advanced Institute of Science and Technology, Daejeon, Republic of Korea; 3 Department of Rheumatology, Hanyang University Hospital for Rheumatic Diseases, Seoul, Republic of Korea; 4 Department of Biology, Kyung Hee University, Seoul, Republic of Korea; 5 Department of Radiology, Hanyang University Hospital, Seoul, Republic of Korea

**Keywords:** Rheumatoid arthritis, Radiographic progression, Bioinformatic analysis, GWAS, Post-GWAS analysis

## Abstract

**Background:**

Radiographic progression is reported to be highly heritable in rheumatoid arthritis (RA). However, previous study using genetic loci showed an insufficient accuracy of prediction for radiographic progression. The aim of this study is to identify a biologically relevant prediction model of radiographic progression in patients with RA using a genome-wide association study (GWAS) combined with bioinformatics analysis.

**Methods:**

We obtained genome-wide single nucleotide polymorphism (SNP) data for 374 Korean patients with RA using Illumina HumanOmni2.5Exome-8 arrays. Radiographic progression was measured using the yearly Sharp/van der Heijde modified score rate, and categorized in no or severe progression. Significant SNPs for severe radiographic progression from GWAS were mapped on the functional genes and reprioritized by post-GWAS analysis. For robust prediction of radiographic progression, tenfold cross-validation using a support vector machine (SVM) classifier was conducted. Accuracy was used for selection of optimal SNPs set in the Hanyang Bae RA cohort. The performance of our final model was compared with that of other models based on GWAS results and SPOT (one of the post-GWAS analyses) using receiver operating characteristic (ROC) curves. The reliability of our model was confirmed using GWAS data of Caucasian patients with RA.

**Results:**

A total of 36,091 significant SNPs with a *p* value <0.05 from GWAS were reprioritized using post-GWAS analysis and approximately 2700 were identified as SNPs related to RA biological features. The best average accuracy of ten groups was 0.6015 with 85 SNPs, and this increased to 0.7481 when combined with clinical information. In comparisons of the performance of the model, the 0.7872 area under the curve (AUC) in our model was superior to that obtained with GWAS (AUC 0.6586, *p* value 8.97 × 10^-5^) or SPOT (AUC 0.7449, *p* value 0.0423). Our model strategy also showed superior prediction accuracy in Caucasian patients with RA compared with GWAS (*p* value 0.0049) and SPOT (*p* value 0.0151).

**Conclusions:**

Using various biological functions of SNPs and repeated machine learning, our model could predict severe radiographic progression relevantly and robustly in patients with RA compared with models using only GWAS results or other post-GWAS tools.

**Electronic supplementary material:**

The online version of this article (doi:10.1186/s13075-017-1414-x) contains supplementary material, which is available to authorized users.

## Background

The marked success of genome-wide association studies (GWAS) has led to the discovery of numerous novel genetic loci. To date, nearly 100 susceptibility loci of rheumatoid arthritis (RA) have been identified [1]. Recently, the role of post-GWAS analysis, which prioritizes GWAS signals by incorporating diverse biological and functional evidence, has been highlighted in the identification of causal loci and for prediction of phenotypic traits [2]. Most genome-wide association loci are in noncoding regions of the genome and might not directly implicate functional variants, whereas the prioritized loci in post-GWAS analysis are biologically relevant variants and more likely to be truly associated with phenotypic traits [2].

Radiographic severity is a pivotal outcome of RA. Prediction of patients who will ultimately develop severe radiographic progression in the initial stage of the disease course is important for better outcomes and necessary for precision medicine. As radiographic severity is reported to be highly heritable (45–58%) [3], genetic loci or genes could be helpful in the prediction of radiographic severity. However, there is currently a lack of genetic information for prediction of radiographic damage. According to a report by van Steenbergen et al., prediction accuracy of severe radiographic progression reached only 62% using a model consisting of 17 known genetic loci from several replication studies and meta-analysis and clinical factors [4].

Therefore, we sought to develop a more accurate and reliable prediction model for radiographic progression using a comprehensive approach consisting of GWAS, post-GWAS analysis, and bioinformatics. We first conducted GWAS of radiographic progression in Korean patients with early RA. Next, single nucleotide polymorphisms (SNPs) conferred by GWAS were mapped and prioritized according to their biological features through a post-GWAS approach and an optimal set of SNPs for prediction of radiographic progression was selected via tenfold cross-validation using a support vector machine (SVM). Next, a prediction model for radiographic progression was generated by the ensemble approach using genetic and clinical factors. Finally, we confirmed the usefulness of post-GWAS prioritization and our model strategy for prediction of radiographic progression in an independent cohort of Caucasian patients with RA.

## Methods

### Patients

All patients fulfilled the 1987 revised American College of Rheumatology criteria [5], and were recruited after providing informed consent and with ethical approval from the Institutional Review Board of Hanyang University Hospital (HYG-14-032-1).

Two cohorts were used to establish the prediction model of severe radiographic progression and their clinical characteristics are shown in Additional file 1: Table S1. First, 374 patients with early RA from the Hanyang Bae RA cohort of Hanyang University Hospital for Rheumatic Diseases [6] with two hand X-rays were included for the initial approach of post-GWAS analysis and construction of a prediction model. Next, reliability of post-GWAS prioritization for prediction of severe radiographic progression was evaluated in 399 patients with RA from the North American Rheumatoid Arthritis Consortium (NARAC) [7] with one hand X-ray per person.

### Radiographic outcome

Radiographic joint damage was measured using the Sharp/Van der Heijde modified score (SHS) from hand radiographs [8]. For analysis of the Hanyang Bae RA cohort with two hand X-rays, the yearly radiographic joint damage rate (ΔSHS/year) was calculated as the difference in SHS between baseline and follow-up radiographs, divided by the duration between the two X-rays. Two independent expert radiologists scored the radiographs and the interclass observer correlation coefficient was 0.89 for the total score. For analysis of the NARAC cohort with one X-ray, the estimated yearly progression rate was calculated (total SHS/disease years at time of X-ray) as explained in a previous study [9]. Trained readers at the Leiden University Medical Center scored radiographs and the intra-observer reliability was 0.99 [10]. Patients with RA were classified into three groups of low, middle, and high tertiles based on their radiographic severity. Only the two groups of low tertile (no progression) and high tertile (severe progression) were used for analysis.

### Genotyping

In the Hanyang Bae RA cohort, genotyping was conducted with Illumina HumanOmni2.5Exome-8 BeadChips at SNP Genetics Inc. (Seoul, South Korea). All subjects were successfully genotyped for >2.5 million markers with reliable genotyping call rates per sample ≥95%. After the quality control, approximately 1.4 million markers with minor allele frequency (MAF) ≥0.5%, genotyping call rate rates per each marker ≥95%, and Hardy Weinberg equilibrium (HWE) >5 × 10^-7^ were used in subsequent analyses. Genetic relationship analysis performed to identify cryptic relatedness among the subjects did not find any duplicates, twins, or first-degree relatives. Principal component (PC) analysis was performed to obtain PCs and assess population stratification among the subjects. We noted that there were no genetic outliers of >6 standard deviations for each of the top ten PCs.

In the NARAC cohort, genotyping was conducted with Illumina BeadChips (HumanHap 550 k) [7]. As reported in a previous study [10], 391,733 SNPs with reliable genotyping success rate (>98%), MAF >0.1%, and >1 × 10^−5^ were used in analyses.

### Genome-wide association study and genome mapping based on functional regions and eQTL data

**Fig. 1:**
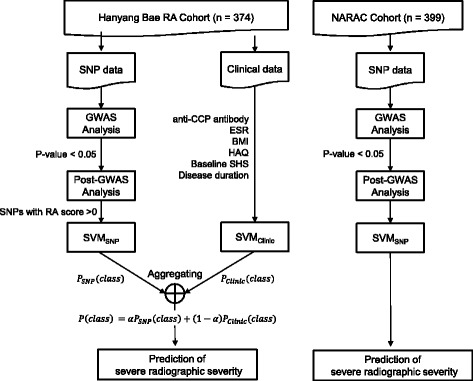
Study workflow. *BMI* body mass index, *CCP* cyclic citrullinated peptide, *ESR* erythrocyte sedimentation rate, *GWAS* genome-wide association studies, *HAQ* health assessment questionnaire, *NARAC* North American Rheumatoid Arthritis Consortium, *SHS* Sharp/Van der Heijde modified score, *SNP* single nucleotide polymorphism, *SVM* support vector machine

A comprehensive approach including GWAS, post-GWAS analysis, repeated machine learning using SVM, and ensemble model was conducted to identify a prediction model for severe radiographic progression. The study workflow is presented in Fig. 1.

First, GWAS was performed in a nested case-control design, yielding genetic predictors for severe radiographic progression. Next, we mapped the statistically significant SNPs (*p* value <0.05 in GWAS analysis) with their biologically related genes based on the functional regions these SNPs map to. For this, we collected functional regions of SNPs from several public databases and obtained a total of 43,011 enhancer regions and associated genes retrieved from the FANTOM5 consortium [11]. A total of 50,900 gene regions, including both coding and intron regions and promoter regions, defined as 2 k bases upstream from the transcription start site, were downloaded from the UCSC table browser [12]. In addition, we collected 4666 miRNA regions from miRbase [13] and their target genes from miRTarBase [14]. Moreover, we assessed cis and trans-expression quantitative trait loci (eQTL) effects by reference to four publicly available datasets [15–18]. We integrated eQTL information tested in peripheral blood mononuclear cells (PBMCs), monocytes, CD4+ T cells, and lymphoblastoids with significance threshold defined in reference papers. When mapping the SNPs, we also considered their proxy SNPs with r2 > 0.8. Reference pair-wise linkage disequilibrium (LD) information was retrieved from HapMap genotype information of Japanese and Han Chinese populations.

### SNP reprioritization based on RA network

**Fig. 2:**
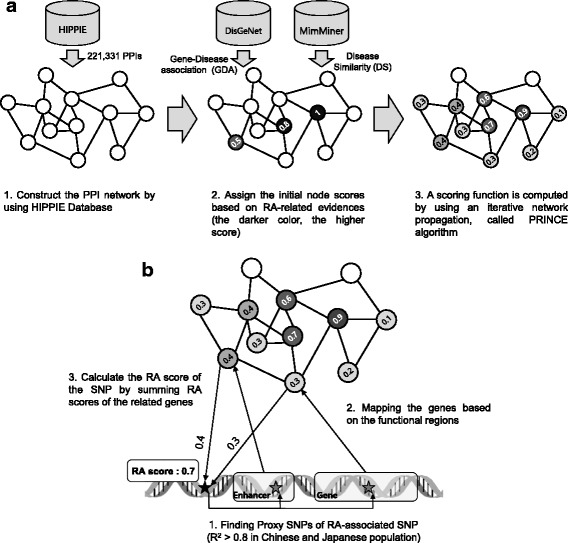
Post-GWAS analysis: construction of an RA network (**a**), mapping statistically significant SNPs with their biological-related genes and calculation of RA correlation score of all genes in the network (**b**). *RA* rheumatoid arthritis, *SNP* single nucleotide polymorphism

We reprioritized the statistically significant SNPs in GWAS based on RA correlation scores of their related genes. To measure the RA correlation of the genes, we first constructed a RA gene network by propagation of prior RA information to their interaction partners (Fig. 2a). To construct the network, we used an integrated gene interaction database called HIPPIE [19], which provided 221,331 interactions between 15,615 genes. We collected prior gene-disease association (GDA) from DisGeNet [20] and disease similarity (DS) from MimMiner [21] to consider not only RA genes, but also genes for RA-related diseases. Next, for a gene v in the Y was assigned as below:

$$ \mathrm{Y}\left(\mathrm{v}\right)=\operatorname{Max}\left(\mathrm{GDA}\_\left(\mathrm{v},\mathrm{d}\right)\times \mathrm{DS}\_\left(\mathrm{d},\mathrm{RA}\right)\right), $$

where d represents all disease that is associated with gene v. With assignment of prior RA information, we propagated the information using the PRINCE method [22] and calculated RA correlation scores of all genes in the network. With the RA correlation scores of genes, we finally reprioritized SNPs by the sum of RA correlation scores of their related genes (Fig. 2b). We also collected SNP sets that were prioritized by *p* value in GWAS analysis and by SPOT analysis [23] for the comparison of prediction powers.

### Prediction model for radiographic severity using ensemble approach

The final prediction model for severe radiographic progression consisted of an ensemble approach that combined two classification models: one was based on the SNPs that we selected and the other was based on clinical information. Each model was constructed by using SVM, which is a supervised machine learning algorithm to classify multiclasses based on a hyperplane that differentiate the classes on the n-dimensional space. We used six clinical predictive factors that we investigated in another study [24]: baseline SHS, disease duration, health assessment questionnaire (HAQ) index, anti-cyclic citrullinated peptide (CCP) antibody, body mass index (BMI), and erythrocyte sedimentation rate (ESR). The final decision for severe radiographic progression was calculated as the weight sum of probabilities in each model.

### Post-GWAS prioritization in an independent cohort of Caucasian patients with RA

To confirm the reliability of post-GWAS prioritization and SVM-based prediction of severe radiographic progression, we conducted GWAS, post-GWAS analysis, and machine learning using SVM in consecutive order. As there was limited clinical information in the NARAC cohort, only SNPs were used for prediction of severe radiographic progression.

### Statistical analysis

The multivariate logistic regression model was used to investigate the association between SNPs and radiographic severity (no progression vs. severe progression) in GWAS, adjusted for anti-CCP antibody positivity, ESR, BMI, HAQ score, baseline SHS, disease duration, and the top ten principal components using PLINK v1.07.

Accuracy is a measure of the proportion of samples that are correctly predicted among all the test samples, and it is easy to intuitively understand the model performance at a glance. Thus, accuracy was used for selection of optimal SNPs set in the Hanyang Bae RA cohort and NARAC cohort, according to the standard method as follows:

$$ \mathrm{Accuracy}=\frac{\sum True\kern0.5em positive+\sum True\kern0.5em negative}{\sum True\kern0.5em population} $$

Classification accuracy is typically not enough information to evaluate the performance of the model. To evaluate the robustness of a model, more performance measures are needed. The area under the curve (AUC) of the receiver operating characteristic (ROC) curve measures the performance of the markers with the total sum of performance at all thresholds. Based on the diversity of populations and the characteristics of SNP markers that should be evaluated with a limited number of samples, it would be more reliable to compare all the performance that the set could have, rather than looking for the best accuracy it could have, with expectations for performance for various unknown samples. In this reason, we further analyzed the performance of the model in the Hanyang Bae RA cohort using AUC according to the standard method as follows:

$$ \mathrm{AUC}=\frac{\sum Rank(pos)-\# pos\times \left(\# pos+1\right)/2}{\# pos+\# neg} $$

Where ∑Rank(pos) means the sum of the ranks of all positively classified examples, #pos means the number of positive examples in the dataset, and #neg means the number of negative examples in the dataset.

## Results

### Findings of GWAS for radiographic progression in Korean RA patients

After quality control, a total of 1,343,748 SNPs were available for comparison in 118 patients with no progression [age 49.5 ± 11.8 (mean ± standard deviation), female = 83.9%] and 120 patients with severe progression [age 47.7 ± 12.6, female = 85.0%] (Additional file 1: Table S1). In the single association analysis, none of the SNPs reached the significance threshold after Bonferroni correction. The SNPs with *p* <1.0 × 10^-3^ and their related genes are listed in Additional file 1: Table S2.

### Optimal SNP set selection using post-GWAS scoring

**Fig. 3:**
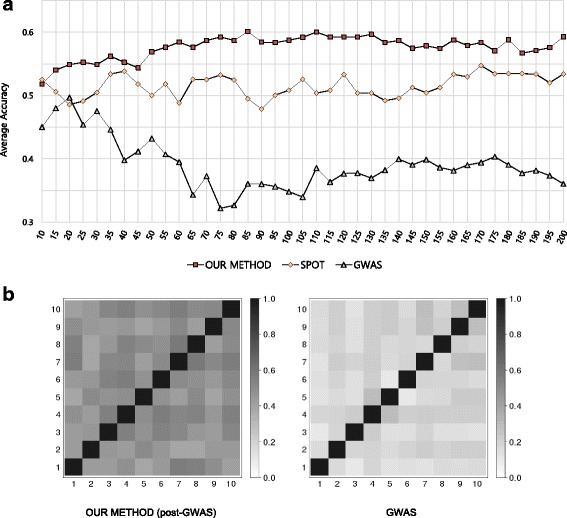
Prediction accuracy of radiographic progression using SNPs obtained via post-GWAS, GWAS, and SPOT analysis: optimal number of SNPs for the prediction model (**a**), and overlapping ratio between 85 SNPs selected by post-GWAS and GWAS analysis (**b**). *GWAS* genome-wide association studies

To determine the optimal number of SNPs for the prediction model, we tested the accuracy of the prediction model by adding 5 SNPs from the top ten scored SNPs. For this, we performed tenfold cross-validation by grouping the patients into ten groups. Of the ten groups, nine groups were used as the training set in GWAS and post-GWAS analysis for selection of the SNPs and construction of a SVM model using radial basis function Kernel. The remaining group was used as a test set and we calculated the average accuracies of ten test sets. Our results showed that the best accuracy was 0.6015 when the top 85 SNPs were used (Fig. 3a). Therefore, we defined the optimal number of SNPs as 85. Our method showed superior accuracy compared with SNPs selected based on p-value of GWAS and by SPOT analysis (*p* value 1.06 × 10^-06^ and 6.25 × 10^-03^, respectively). The list of 85 SNPs and their related genes are described in Additional file 1: Table S3.

Interestingly, SNPs that had low *p* values in GWAS analysis showed the lowest accuracy. To investigate further, we compared the overlapping ratio of the top 85 SNPs selected by different methods between ten training sets (Fig. 3b). The results showed that the overlap ratio between SNPs selected by low *p* value was only 0.2403, whereas the overlap ratio of our method was more than two times higher (0.5627). It seems that the SNPs selected in each training set by tenfold cross-validation based on GWAS *p* value are likely to be biased in each training sample itself and could cause lower overlap ratio between groups. On other hand, post-GWAS analysis which integrated the biological meaning to the analysis is less likely to select the SNPs that were biased in the sample group, unlike the GWAS which depends only on the simple *p* value.

### Comparison of the prediction accuracy among different models

**Fig. 4:**
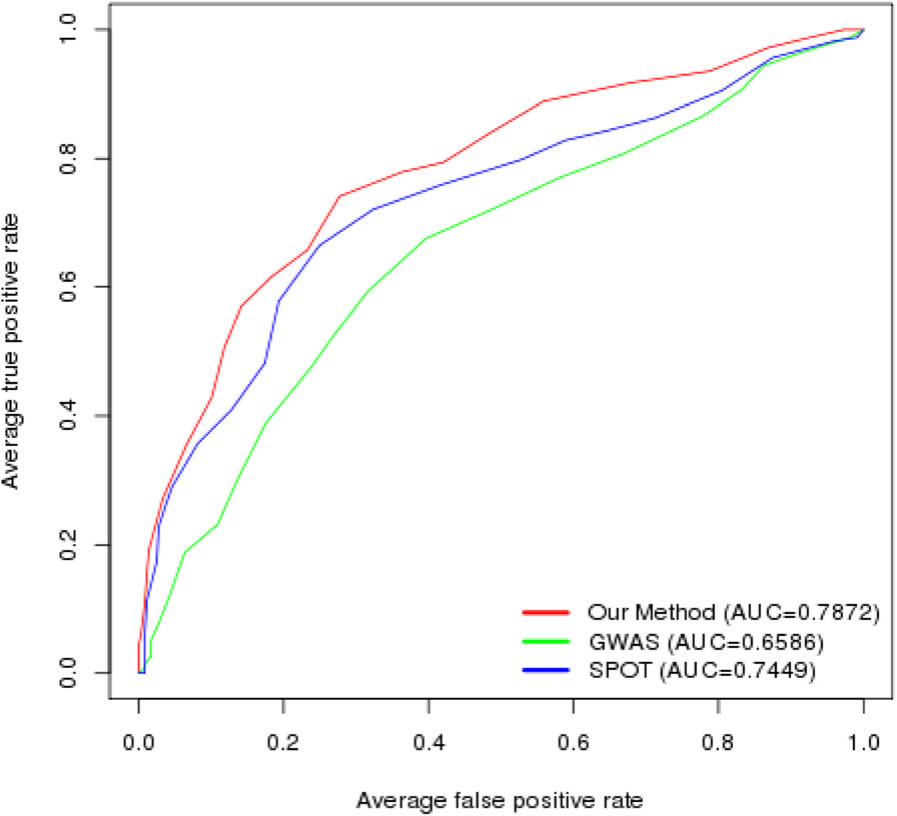
Comparison of prediction accuracy of our ensemble model with other methods in Korean patients with RA. AUC area under the curve, *GWAS* genome-wide association studies

The final ensemble model was composed of two different SVM models: one is based on 85 SNPs selected by post-GWAS analysis and the other is based on information on six clinical factors. We calculated a weighted sum of probabilities of these two models to predict severe radiographic progression. The best average accuracy of our model was 0.7481 with 0.27 of weight to SVM model using SNPs (Additional file 2: Figure S1). In the process of optimizing the weight, all tenfold cross-validation tests were performed on the test set, to avoid overfitting as much as possible. We compared the ROC curve of our ensemble model with other ensemble models that used 85 SNPs selected by GWAS *p* value or by SPOT analysis as well as clinical information (Fig. 4). The AUC of our model was 0.7872 (sensitivity 0.7644, specificity 0.7318, and positive predictive value 0.7445, Additional file 1: Table S4), which was significantly better than that of the ensemble model with GWAS (8.97X10E-5) and SPOT (0.0423).

### Reliability of post-GWAS prioritization in the independent cohort

**Fig. 5:**
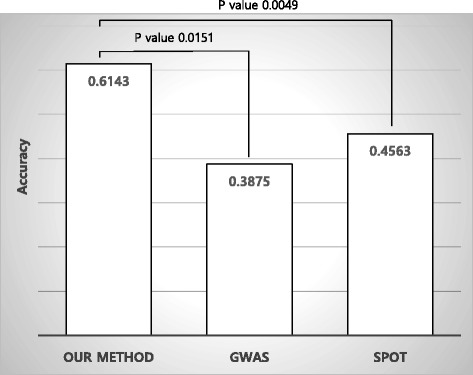
Comparison of prediction accuracy of our model with other methods in an independent Caucasian cohort. *GWAS* genome-wide association studies

By applying the same methods of post-GWAS prioritization and tenfold cross-validation using SVM to the NARAC cohort (68 patients with no progression and 86 patients with severe progression), we were able to confirm that the SNPs selected by post-GWAS analysis were more accurate than those selected by statistical significance in GWAS for prediction of severe radiographic progression. In the NARAC cohort the average accuracy was 0.6143 with SNPs selected by post-GWAS analysis, which was superior to that using SNPs selected by statistical significance in GWAS (average accuracy 0.3875) or by SPOT analysis (average accuracy 0.4563) (Fig. 5).

After quality control, a total of 1,343,748 SNPs were available for comparison in 118 patients with no progression [age 49.5 ± 11.8 (mean ± standard deviation), female = 83.9%] and 120 patients with severe progression [age 47.7 ± 12.6, female = 85.0%] (Additional file 1: Table S1).

## Discussion

As hypothesized in this study, our new model allowed us to conduct more relevant and robust prediction of radiographic severity in RA. In short, using post-GWAS analysis we identified biologically relevant SNPs related to RA progression in patients with early RA. Our final model composed of SNPs combined with clinical factors could satisfactorily discriminate severe progression from the absence of progression, showing an average AUC of 0.78 in tenfold cross-validation. This result was superior to those obtained using data from GWAS (AUC = 0.59) or SPOT (AUC = 0.67), one of the methods of post-GWAS analysis. The superior effectiveness of our prediction model was also successfully reproduced in an independent cohort.

We initially thought that biological function-enriched prediction of radiographic severity would overcome the overfitting effect although the prediction accuracy would be similar to that using only GWAS results. Interestingly, however, the prediction accuracy was also improved compared with that using data of GWAS. Selection of biologically relevant variants based on post-GWAS analysis, in addition to *p* value in GWAS, and use of a machine learning algorithm such as SVM enabled more accurate and robust prediction of radiographic severity despite the limited sample size.

Regarding post-GWAS analysis, there have been examples of effective integration of biological database information of SNPs with GWAS results to identify causal SNPs in colorectal cancer [25] and chronic lymphocytic lymphoma [26]. We used information on various functional regions associated with SNPs and related genes such as enhancer region, mRNA, promoter region, miRNA region, and posttranscriptional modification (PTM), in addition to expression of quantitative traits, and gave a higher priority to SNPs with greater involvement with these genes. Thus, SNPs with higher biological relevance obtained a higher reprioritization score and might be used in prediction of radiographic severity.

The SVM algorithm also contributed to increased prediction accuracy. It is one of the popular supervised learning techniques in classification. In the SVM algorithm, each patient is represented in n-dimensional space where n is the number of SNPs. After that, it finds a hyperplane that can separate patients’ classes with maximum margin. We also used Kernel trick that mapped original dimensional space into a much higher-dimensional space. It can help to do a nonlinear classification more efficiently. This learning machine technique could discover new patterns for input features via investigation of complex relationships among SNPs, and thus increase the explanation power for prediction of radiographic severity in RA [27]. Many examples of outcome prediction with high predictive accuracy using SVM algorithms have been reported, such as in breast cancer [28], nasopharyngeal carcinoma [29], and severe radiation-induced pneumonitis in lung cancer [30–32]. Similarly, we could predict severe radiographic progression with high predictive accuracy via a SVM-based ensemble model that integrated multidimensional SNP data and clinical factors.

It is interesting that our model was superior to SPOT, which is also a method of SNP prioritization [23]. However, there were some differences between our SNP prioritization method and SPOT. Information on functional properties used in annotation was not the same; our method used more varied biological information related to SNPs and genes including transcription factor binding sites, micro RNA regions, PTM, and eQTL. An eQTL study was able to explain the functional basis of up to 50% of SNPs related to immune-mediated disease [33] and therefore might be very useful in predicting the outcome of RA. Another important difference is the characteristics of the network used for scoring. In contrast to SPOT, we used a disease-specific gene database during construction of the network based on the concept that a RA susceptibility gene is also associated with phenotype. This is the first RA-specific network constructed based on network propagation and might give more accurate and stable relationship information to reprioritize the SNPs conferred by GWAS.

This study has some limitations. First, the sample size used in the analysis was small, which could lead to lower predictive accuracy of GWAS. However, as we applied the results of GWAS to post-GWAS analysis and tenfold cross-validation we could achieve higher predictive accuracy of radiographic progression and robustness of top SNPs in each of the ten groups. This meant that post-GWAS could take advantage of the small sample size of subjects in contrast to GWAS, which needs numerous samples to identify disease-specific loci. Second, we did not use the 85 SNPs selected in the Korean cohort in the analysis of the Caucasian cohort. Rather, we reproduced all courses of analysis from GWAS to using the SVM classifier in the Caucasian cohort to show the advantage of a post-GWAS approach over GWAS as a method of prediction. When we validated the final SNPs from the Hanyang Bae RA cohort in the NARAC cohort, the performance of the model using the final SNPs was unsatisfactory. Among the 85 SNPs, 72 SNPs were identified in the NARAC cohort and the accuracy (standard deviation) of the model was 0.5062 (0.1239) and the AUC was 0.4739 (Additional file 2: Figure S2). It seems that the same SNPs are not useful across ethnic groups for many reasons, such as ethnicity-specific SNPs or different allele frequency, or linkage disequilibrium.

## Conclusions

We demonstrated that biologically relevant SNPs could provide more accurate and robust prediction of severe radiographic progression in Korean and Caucasian cohorts. Biologically relevant prediction of radiographic progression was possible through a bioinformatics approach including post-GWAS, which was conducted with functional annotation of the genome gathered from GWA studies, a RA network with propagation, and machine learning algorithm. This approach worked better than the GWAS approach alone. SNPs and genes selected in this approach could be targets for further functional studies and might be a basis of individual precision medicine.

## Additional files

Additional file 1: Table S1. Characteristics of study populations. **Table S2.** GWAS results for severe radiographic progression (*p* <1.0 × 10–3). **Table S3.** List of the top 85 SNPs and their related genes selected by a post-GWAS approach. **Table S4** Sensitivity, specificity, and positive predictive value of the final models. (DOCX 77 kb)

Additional file 2: Figure S1. The weighted sum of probabilities in each model. **Figure S2.** Receiver operating characteristic (ROC) curve for the result of replication of the final SNPs in the NARAC cohort. (PPTX 207 kb)

## Abbreviations

AUC: Area under the curve
BMI: Body mass index
CCP: Cyclic citrullinated peptide
eQTL: Expression quantitative trait loci
ESR: Erythrocyte sedimentation rate
GWAS: Genome-wide association studies
HAQ: health assessment questionnaire
HWE: Hardy Weinberg equilibrium
MAF: Minor allele frequency
NARAC: North American Rheumatoid Arthritis Consortium
PC: Principal component
PTM: posttranscriptional modification
RA: Rheumatoid arthritis
ROC: Receiver operating characteristic
SHS: Sharp/Van der Heijde modified score
SNP: Single nucleotide polymorphisms
SVM: Support vector machine
