# Supplementary material for: Biological function integrated prediction of severe radiographic progression in rheumatoid arthritis: a nested case control study
Source: Arthritis Res Ther. 2017 Oct 25;19:244. doi: 10.1186/s13075-017-1414-x (PMC5655942; doi:10.1186/s13075-017-1414-x)
Supplement: Supplementary file 1 — Characteristics of study populations. Table S2. GWAS results for severe radiographic progression (p <1.0 × 10^–3^). Table S3. List of the top 85 SNPs and their related genes selected by a post-GWAS approach. Table S4 Sensitivity, specificity, and positive predictive value of the final models. (DOCX 77 kb) [file 13075_2017_1414_MOESM1_ESM.docx]

Table S1 Characteristics of study populations

|  | Hanyang Bae RA Cohort | | | | NARAC Cohort | | | | p-value** |
| --- | --- | --- | --- | --- | --- | --- | --- | --- | --- |
|  | Total | No progression  (Control) | Severe progression  (Case) | p-value* | Total | No progression  (Control) | Severe progression  (Case) | p-value* |  |
| Samples, N | 238 | 118 | 120 |  | 154 | 68 | 86 |  |  |
| Age at symptom onset,  mean ± SD (years) | 48.6±12.2 | 49.5±11.8 | 47.7±12.6 | 0.2569 | 41.4±11.8 | 40.4±10.1 | 42.3±12.9 | 0.3061 | 0.5642 |
| Sex, female, N (%) | 201 (84.5%) | 99 (83.9%) | 102 (85%) | 0.9556 | 109 (70.8%) | 48 (70.6%) | 61 (70.9%) | 1.0000 | 1.0000 |
| Disease duration,  mean ± SD (years) | 3.8±2.7 | 3.0±2.2 | 4.7±3.0 | <.0001 | 10.3±8.5 | 10.9±8.2 | 9.8±8.7 | 0.4005 | 0.1765 |
| Anti-CCP antibody, N (%) | 209 (87.8%) | 98 (83.1%) | 111 (92.5%) | 0.0424 | 154 (100%) | 68 (100%) | 86 (100%) | - | 0.0266 |
| Baseline SHS,  mean ± SD | 6.4±14.3 | 2.2±9.6 | 10.6±16.9 | <.0001 | 37.1±47.7 | 2.4±2.7 | 64.5±48.6 | <.0001 | <.0001 |
| ΔSHS/year, mean ± SD | 3.7±5.4 | 0.0±0.0 | 7.3±5.7 | <.0001 | 4.9±6.2 | 0.2±0.2 | 8.7±6.1 | <.0001 | <.0001 |

* represents the statistical difference between no and severe progression in each cohort and ** represents the statistical difference between Hanyang Bae RA and NARAC Cohort.

Hanyang Bae RA Cohort: Among the 2,106 patients enrolled from 2001 to 2013, 374 patients with early RA were enrolled in this study. 118 patients with no progression and 120 patients with severe progression were used for the actual analysis, except for 136 patients with mild progression or insufficient clinical data.

NARAC Cohort: 68 patients with no progression and 86 patients with severe progression were used for the actual analysis, except for 245 patients with mild progression.

Table S2 GWAS results for severe radiographic progression (P < 1.0x10^-3^)

| SNP | CHR | Allele | OR | P | Nearest gene(s) |
| --- | --- | --- | --- | --- | --- |
| kgp12350297 | 18 | T | 4.467 | 0.000004606 | MIB1/GATA6 |
| rs428225 | 2 | C | 0.2068 | 0.000007527 | TGFA |
| kgp10833009 | 18 | A | 0.2293 | 0.00001439 | GALR1/SALL3 |
| kgp12470234 | 8 | C | 0.3168 | 0.00001623 | CSGALNACT1/INTS10 |
| kgp5871836 | 19 | G | 0.1142 | 0.00001786 | LRRC4B/SNAR-F |
| rs9378774 | 6 | G | 3.528 | 0.00002085 | DUSP22/IRF4 |
| kgp4312507 | 6 | G | 3.489 | 0.00002187 | DUSP22/IRF4 |
| kgp9661503 | 14 | A | 0.000002583 | 0.00003127 | PTCSC3/MBIP |
| rs507095 | 16 | G | 2.914 | 0.0000344 | SOX8/SSTR5-AS1 |
| rs458664 | 6 | A | 3.331 | 0.00003559 | FAM65B/CMAHP |
| rs12531117 | 7 | G | 0.3052 | 0.00004247 | CNTNAP2 |
| rs7827153 | 8 | T | 2.981 | 0.00004469 | CSGALNACT1/INTS10 |
| rs9838423 | 3 | A | 3.2 | 0.00004575 | CNTN6/CNTN4 |
| kgp11105201 | 18 | C | 3.193 | 0.00005044 | DLGAP1 |
| kgp10085446 | 8 | G | 0.1751 | 0.00006042 | LINC00535 |
| kgp10598699 | 3 | T | 2.646 | 0.00006443 | LINC00693/RBMS3 |
| kgp7071537 | 9 | T | 4.44 | 0.00006765 | ADAMTSL1 |
| kgp12038314 | 2 | C | 0.2596 | 0.00006814 | TGFA |
| rs2826638 | 21 | C | 3.513 | 0.00007137 | LINC00320/NCAM2 |
| rs1149718 | 10 | C | 0.116 | 0.00007251 | ZNF503-AS1 |
| kgp4815736 | 8 | A | 2.913 | 0.00007691 | LOC100130964 |
| kgp7414736 | 3 | A | 0.2743 | 0.00008007 | PHLDB2/PLCXD2 |
| kgp7200995 | 12 | C | 0.3703 | 0.00008027 | KSR2 |
| kgp824440 | 8 | T | 0.1709 | 0.00008638 | LINC00535 |
| kgp3182123 | 1 | A | 4.415 | 0.00009129 | LINC00210/RRP15 |
| kgp2460881 | 2 | T | 0.2168 | 0.00009366 | IMMT |
| rs11220329 | 11 | C | 0.3001 | 0.00009688 | CDON |
| rs7085145 | 10 | G | 0.3701 | 0.00009778 | APBB1IP |
| kgp442024 | 9 | C | 6.682 | 0.0001002 | ADAMTSL1 |
| rs406161 | 16 | C | 2.746 | 0.0001008 | SOX8/SSTR5-AS1 |
| rs674119 | 18 | G | 0.2537 | 0.0001067 | GALR1/SALL3 |
| rs2902113 | 1 | A | 0.3305 | 0.0001069 | FAM5B/SEC16B |
| kgp11690227 | 18 | C | 3.084 | 0.0001128 | DLGAP1 |
| rs11207153 | 1 | G | 0.3004 | 0.0001151 | DAB1 |
| rs7236680 | 18 | A | 0.213 | 0.0001151 | GALR1/SALL3 |
| kgp1861694 | 13 | T | 0.3913 | 0.0001153 | GSX1/PDX1 |
| kgp186908 | 14 | T | 0.3109 | 0.0001162 | NONE/FLRT2 |
| rs4726968 | 7 | T | 0.3359 | 0.0001174 | CNTNAP2 |
| kgp12363483 | 15 | T | 4.66 | 0.0001188 | RGMA/MCTP2 |
| kgp5995137 | 10 | C | 0.3449 | 0.0001199 | AFAP1L2 |
| kgp5717686 | 13 | T | 3.045 | 0.0001215 | LCP1 |
| rs2897394 | 2 | A | 2.701 | 0.0001256 | HS6ST1/LOC389033 |
| rs10241921 | 7 | A | 3.945 | 0.0001289 | POT1/GRM8 |
| kgp13027017 | 11 | G | 0.3652 | 0.0001312 | NELL1 |
| rs898927 | 14 | C | 0.3421 | 0.0001357 | LOC100129345/C14orf64 |
| rs10049987 | 4 | A | 2.674 | 0.0001377 | COL25A1 |
| kgp3938028 | 4 | A | 2.756 | 0.0001382 | SLC10A7/POU4F2 |
| rs16835308 | 1 | C | 0.2436 | 0.0001385 | S100A7A/S100A7L2 |
| rs12696521 | 3 | G | 0.2419 | 0.0001401 | CHRD/EPHB3 |
| kgp10453381 | 2 | G | 0.2126 | 0.0001402 | MIR4268/EPHA4 |
| rs9658625 | 12 | G | 3.407 | 0.0001412 | SOAT2 |
| kgp1313235 | 1 | A | 0.2772 | 0.0001415 | S100A7 |
| rs448653 | 16 | C | 3.256 | 0.0001425 | SOX8/SSTR5-AS1 |
| kgp9324935 | 18 | G | 3.586 | 0.0001431 | CCDC178 |
| rs7284476 | 22 | G | 0.3612 | 0.000144 | TRIOBP |
| kgp5248164 | 5 | A | 0.387 | 0.0001448 | LHFPL2/ARSB |
| kgp1677070 | 11 | T | 0.2885 | 0.0001448 | RSF1 |
| kgp22753614 | 23 | C | 2.482 | 0.000145 |  |
| rs2042753 | 15 | A | 0.3516 | 0.0001459 | LOC503519/GABRB3 |
| kgp3718245 | 14 | T | 0.3346 | 0.0001489 | SEL1L/NONE |
| kgp9324961 | 12 | T | 0.3828 | 0.0001542 | TSPAN9 |
| rs7778404 | 7 | G | 0.1478 | 0.0001584 | CHCHD3/,EXOC4 |
| kgp6723290 | 14 | A | 0.3324 | 0.000159 | SLC25A47 |
| rs6749090 | 2 | T | 0.3346 | 0.0001595 | IMMT |
| kgp6576387 | 4 | T | 2.722 | 0.0001608 | COL25A1 |
| rs6982077 | 8 | C | 0.3133 | 0.0001611 | LINC00293/LOC100287846 |
| kgp10658954 | 10 | A | 0.08593 | 0.0001622 | DUPD1/DUSP13 |
| kgp3717614 | 3 | T | 0.3234 | 0.000163 | TBC1D5 |
| kgp6803940 | 1 | T | 2.768 | 0.0001662 | RGS21/RGS1 |
| kgp4558063 | 9 | A | 0.1269 | 0.000168 | FAM214B/UNC13B |
| kgp10450961 | 20 | C | 2.646 | 0.0001682 | PLCB1 |
| rs10239446 | 7 | A | 0.3647 | 0.0001695 | PCLO/SEMA3E |
| rs10786620 | 10 | T | 0.2903 | 0.0001697 | TLX1NB |
| rs5927925 | 23 | T | 0.3422 | 0.0001719 |  |
| kgp7585694 | 3 | A | 3.934 | 0.0001779 | PLSCR5/ZIC4 |
| rs6959358 | 7 | G | 2.704 | 0.0001805 | SLC25A13 |
| kgp2733042 | 5 | A | 0.2105 | 0.0001806 | RAB3C |
| kgp5037465 | 4 | A | 0.3013 | 0.0001833 | PRKG2/RASGEF1B |
| kgp518307 | 11 | T | 4.145 | 0.0001884 | SBF2-AS1 |
| kgp8620115 | 15 | T | 7.283 | 0.0001893 | NTRK3-AS1/MRPL46 |
| kgp22750511 | 23 | G | 2.435 | 0.0001919 |  |
| kgp7931552 | 18 | A | 2.929 | 0.0001944 | TNFRSF11A |
| kgp9397699 | 19 | A | 4.802 | 0.0001962 | CALM3/PTGIR |
| rs9877898 | 3 | C | 2.954 | 0.0001964 | LPP |
| kgp9859963 | 11 | C | 0.3221 | 0.000197 | FOLH1/LOC440040 |
| kgp6452642 | 2 | G | 0.04895 | 0.0001978 | NONE/SLC39A10 |
| kgp10087458 | 8 | G | 2.498 | 0.0001979 | LINC00535 |
| rs4686986 | 3 | T | 3.757 | 0.0002027 | LPP |
| rs6444106 | 3 | A | 2.669 | 0.0002044 | ETV5 |
| kgp3529864 | 18 | G | 2.695 | 0.0002052 | MIB1/GATA6 |
| kgp11662788 | 7 | C | 0.3472 | 0.000207 | CNTNAP2 |
| kgp10616425 | 13 | T | 0.2348 | 0.000208 | LINC00353/LINC00559 |
| rs4691702 | 4 | C | 3.716 | 0.0002102 | RAPGEF2/FSTL5 |
| kgp7466047 | 18 | G | 2.987 | 0.000211 | DLGAP1 |
| kgp21737630 | 10 | A | 0.2477 | 0.0002122 | TMEM26/C10orf107 |
| kgp6428417 | 8 | T | 0.3208 | 0.0002158 | LINC00535 |
| rs4430266 | 23 | C | 2.412 | 0.000218 |  |
| rs16906158 | 11 | C | 0.2352 | 0.0002246 | FOLH1 |
| rs3778638 | 6 | A | 0.07988 | 0.0002252 | PSORS1C1 |
| rs12619211 | 2 | G | 0.3601 | 0.0002277 | NRXN1/ASB3 |

Table S3 List of the top 85 SNPs and their related genes selected by a post-GWAS approach

| **Rank** | **SNP** | **Nearest gene** | **Related sequence type** | **Functional gene** |
| --- | --- | --- | --- | --- |
| 1 | rs1837 | PHF19 | eQTL | C5 |
|  |  |  | eQTL | TRAF1 |
|  |  |  | eQTL | PSMD5 |
|  |  |  | eQTL | PHF19 |
|  |  |  | mRNA | PHF19 |
|  |  |  | mRNA | FBXW2 |
|  |  |  | Promoter | PSMD5 |
| 2 | rs411337 | TNXB | eQTL | HLA-DQB1 |
|  |  |  | eQTL | HSPA1B |
|  |  |  | eQTL | HSPA1A |
|  |  |  | eQTL | RDBP |
|  |  |  | eQTL | HSPA1L |
|  |  |  | eQTL | SKIV2L |
|  |  |  | eQTL | HLA-DRB5 |
|  |  |  | eQTL | LSM2 |
|  |  |  | mRNA | TNXB |
|  |  |  | mRNA | SKIV2L |
|  |  |  | mRNA | ATF6B |
|  |  |  | mRNA | FKBPL |
|  |  |  | Promoter | ATF6B |
| 3 | rs9263800 | POU5F1 | eQTL | HLA-C |
|  |  |  | eQTL | HLA-C |
|  |  |  | eQTL | HLA-C |
|  |  |  | eQTL | HLA-C |
|  |  |  | eQTL | HLA-C |
|  |  |  | eQTL | HLA-C |
|  |  |  | eQTL | HLA-C |
|  |  |  | eQTL | HLA-C |
|  |  |  | eQTL | MICB |
|  |  |  | eQTL | VARS2 |
|  |  |  | eQTL | TCF19 |
|  |  |  | mRNA | POU5F1 |
|  |  |  | mRNA | CCHCR1 |
|  |  |  | mRNA | PSORS1C2 |
|  |  |  | mRNA | TCF19 |
|  |  |  | Promoter | POU5F1 |
|  |  |  | Promoter | CCHCR1 |
|  |  |  | Promoter | PSORS1C2 |
|  |  |  | Promoter | TCF19 |
| 4 | rs2071543 | PSMB8 | Enhancer | RGL2 |
|  |  |  | Enhancer | TAPBP |
|  |  |  | Enhancer | B3GALT4 |
|  |  |  | Enhancer | HLA-DRA |
|  |  |  | Enhancer | HLA-DPA1 |
|  |  |  | Enhancer | BRD2 |
|  |  |  | Enhancer | PSMB8 |
|  |  |  | Enhancer | PSMB9 |
|  |  |  | Enhancer | HLA-DMB |
|  |  |  | Enhancer | TAP1 |
|  |  |  | Enhancer | TAP2 |
|  |  |  | Enhancer | HLA-DRB5 |
|  |  |  | eQTL | HLA-DOA |
|  |  |  | eQTL | PSMB8 |
|  |  |  | eQTL | PSMB9 |
|  |  |  | eQTL | TAP2 |
|  |  |  | mRNA | TAP1 |
| 5 | rs9357155 | PSMB8 | Enhancer | RGL2 |
|  |  |  | Enhancer | TAPBP |
|  |  |  | Enhancer | B3GALT4 |
|  |  |  | Enhancer | HLA-DRA |
|  |  |  | Enhancer | HLA-DPA1 |
|  |  |  | Enhancer | BRD2 |
|  |  |  | Enhancer | PSMB8 |
|  |  |  | Enhancer | PSMB9 |
|  |  |  | Enhancer | HLA-DMB |
|  |  |  | Enhancer | TAP1 |
|  |  |  | Enhancer | TAP2 |
|  |  |  | Enhancer | HLA-DRB5 |
|  |  |  | eQTL | HLA-DOA |
|  |  |  | eQTL | PSMB8 |
|  |  |  | eQTL | PSMB9 |
|  |  |  | eQTL | TAP2 |
|  |  |  | mRNA | TAP1 |
| 6 | rs2044102 | CARD14 | eQTL | GAA |
|  |  |  | eQTL | SGSH |
|  |  |  | mRNA | CARD14 |
|  |  |  | Promoter | CARD14 |
| 7 | rs12722588 | IL2RA | mRNA | IL2RA |
| 8 | rs3130100 | TAPBP | Enhancer | TAPBP |
|  |  |  | Enhancer | PHF1 |
|  |  |  | Enhancer | B3GALT4 |
|  |  |  | Enhancer | BRD2 |
|  |  |  | Enhancer | PSMB8 |
|  |  |  | Enhancer | PSMB9 |
|  |  |  | Enhancer | RPS18 |
|  |  |  | Enhancer | LEMD2 |
|  |  |  | eQTL | TAPBP |
|  |  |  | eQTL | CUTA |
|  |  |  | eQTL | B3GALT4 |
|  |  |  | eQTL | HLA-DPB1 |
|  |  |  | eQTL | RING1 |
|  |  |  | eQTL | VPS52 |
|  |  |  | eQTL | HSD17B8 |
|  |  |  | mRNA | ZBTB22 |
|  |  |  | mRNA | PFDN6 |
|  |  |  | mRNA | WDR46 |
|  |  |  | mRNA | TAPBP |
|  |  |  | mRNA | RPS18 |
|  |  |  | mRNA | VPS52 |
|  |  |  | Promoter | PFDN6 |
|  |  |  | Promoter | WDR46 |
|  |  |  | Promoter | TAPBP |
|  |  |  | Promoter | VPS52 |
| 9 | rs17409828 | FRAS1 | eQTL | ANXA3 |
|  |  |  | mRNA | FRAS1 |
| 10 | rs32490 | RNU6ATAC2P | eQTL | ANKRD55 |
| 11 | rs10065841 | CTD-2227I18.1 | eQTL | ANKRD55 |
| 12 | rs10045084 | CTD-2227I18.1 | eQTL | ANKRD55 |
| 13 | rs154828 | RNU6ATAC2P | eQTL | ANKRD55 |
| 14 | rs4699912 | RNU6ATAC2P | eQTL | ANKRD55 |
| 15 | rs2929660 | RP11-326N17.1 | Enhancer | RASGRP1 |
| 16 | rs17030 | AP-2/Zfp691 | eQTL | TNFSF14 |
|  |  |  | mRNA | C3 |
| 17 | rs2039223 | F11R | eQTL | HCRT |
|  |  |  | eQTL | USF1 |
|  |  |  | eQTL | PPOX |
|  |  |  | eQTL | ARHGAP30 |
|  |  |  | eQTL | ITLN2 |
|  |  |  | eQTL | LY9 |
|  |  |  | eQTL | F11R |
|  |  |  | mRNA | F11R |
| 18 | rs2523537 | U6 | eQTL | LTA |
|  |  |  | eQTL | BAG6 |
|  |  |  | eQTL | AIF1 |
|  |  |  | eQTL | MICB |
|  |  |  | eQTL | HCP5 |
| 19 | rs12118313 | ARHGAP30 | eQTL | HCRT |
|  |  |  | eQTL | USF1 |
|  |  |  | eQTL | PPOX |
|  |  |  | eQTL | ARHGAP30 |
|  |  |  | eQTL | ITLN2 |
|  |  |  | eQTL | F11R |
|  |  |  | mRNA | ARHGAP30 |
| 20 | rs12129787 | FCGR2A | eQTL | FCGR3B |
|  |  |  | eQTL | FCGR2A |
|  |  |  | mRNA | HSPA6 |
| 21 | rs7323390 | FCGR2A | Enhancer | GPR183 |
|  |  |  | Enhancer | GPR18 |
|  |  |  | Enhancer | CLYBL |
|  |  |  | Enhancer | DOCK9 |
|  |  |  | mRNA | GPR183 |
|  |  |  | mRNA | UBAC2 |
| 22 | rs2281091 | Foxc1/Pbx3 | eQTL | IL2RB |
|  |  |  | mRNA | TMPRSS6 |
| 23 | rs2235325 | SMC3 | eQTL | IL2RB |
|  |  |  | mRNA | TMPRSS6 |
| 24 | rs3132935 | NOTCH4 | eQTL | HLA-DRA |
|  |  |  | eQTL | SKIV2L |
|  |  |  | eQTL | AGPAT1 |
|  |  |  | mRNA | NOTCH4 |
| 25 | rs9263719 | PSORS1C1 | eQTL | HLA-C |
|  |  |  | eQTL | HLA-C |
|  |  |  | eQTL | HLA-C |
|  |  |  | eQTL | HLA-C |
|  |  |  | eQTL | HLA-C |
|  |  |  | eQTL | HLA-C |
|  |  |  | eQTL | HLA-C |
|  |  |  | eQTL | HLA-C |
| 26 | rs16822604 | IRS1 | eQTL | IRS1 |
|  |  |  | mRNA | IRS1 |
| 27 | rs213204 | RPS18/VPS52 | eQTL | TAPBP |
|  |  |  | eQTL | CUTA |
|  |  |  | eQTL | B3GALT4 |
|  |  |  | eQTL | HLA-DPB1 |
|  |  |  | eQTL | RING1 |
|  |  |  | eQTL | VPS52 |
|  |  |  | eQTL | HSD17B8 |
|  |  |  | mRNA | ZBTB22 |
|  |  |  | mRNA | PFDN6 |
|  |  |  | mRNA | WDR46 |
|  |  |  | mRNA | TAPBP |
|  |  |  | mRNA | VPS52 |
|  |  |  | Promoter | PFDN6 |
|  |  |  | Promoter | WDR46 |
|  |  |  | Promoter | TAPBP |
| 28 | rs3788368 | SUSD2 | eQTL | ADORA2A |
|  |  |  | eQTL | GSTT1 |
|  |  |  | eQTL | SUSD2 |
|  |  |  | mRNA | SUSD2 |
|  |  |  | mRNA | CABIN1 |
| 29 | rs10192014 | BHLHE40 | eQTL | IL1RN |
|  |  |  | eQTL | PSD4 |
| 30 | rs315919 | IL1RN | eQTL | IL1RN |
|  |  |  | eQTL | PSD4 |
|  |  |  | mRNA | IL1RN |
| 31 | rs3130014 | DAXX | eQTL | TAPBP |
|  |  |  | eQTL | CUTA |
|  |  |  | eQTL | B3GALT4 |
|  |  |  | eQTL | HLA-DPB1 |
|  |  |  | eQTL | VPS52 |
|  |  |  | mRNA | ZBTB22 |
|  |  |  | mRNA | PFDN6 |
|  |  |  | mRNA | TAPBP |
|  |  |  | Promoter | WDR46 |
|  |  |  | Promoter | TAPBP |
| 32 | rs804267 | NEIL2 | eQTL | CTSB |
|  |  |  | eQTL | FDFT1 |
|  |  |  | eQTL | BLK |
|  |  |  | eQTL | MTMR9 |
|  |  |  | mRNA | NEIL2 |
| 33 | rs982764 | FAS | eQTL | FAS |
|  |  |  | eQTL | FASN |
|  |  |  | eQTL | ACTA2 |
|  |  |  | eQTL | STAMBPL1 |
|  |  |  | mRNA | FAS |
|  |  |  | mRNA | ACTA2 |
| 34 | rs4406737 | FAS | eQTL | FAS |
|  |  |  | eQTL | FASN |
|  |  |  | eQTL | ACTA2 |
|  |  |  | eQTL | STAMBPL1 |
|  |  |  | mRNA | FAS |
|  |  |  | mRNA | ACTA2 |
|  |  |  | Promoter | FAS |
|  |  |  | Promoter | ACTA2 |
| 35 | rs4934433 | ACTA2-FAS | eQTL | FAS |
|  |  |  | eQTL | FASN |
|  |  |  | eQTL | ACTA2 |
|  |  |  | eQTL | STAMBPL1 |
|  |  |  | mRNA | FAS |
|  |  |  | mRNA | ACTA2 |
|  |  |  | Promoter | FAS |
|  |  |  | Promoter | ACTA2 |
| 36 | rs213210 | MIR219-1 | eQTL | TAPBP |
|  |  |  | eQTL | B3GALT4 |
|  |  |  | eQTL | HLA-DPB1 |
|  |  |  | eQTL | HLA-DOA |
|  |  |  | eQTL | HLA-DPA1 |
|  |  |  | eQTL | HSD17B8 |
|  |  |  | mRNA | COL11A2 |
|  |  |  | mRNA | HSD17B8 |
| 37 | rs2072634 | CFB | eQTL | HSPA1A |
|  |  |  | eQTL | HSPA1B |
|  |  |  | eQTL | RDBP |
|  |  |  | eQTL | SKIV2L |
|  |  |  | eQTL | LY6G6F |
|  |  |  | mRNA | C2 |
|  |  |  | mRNA | RDBP |
| 38 | rs4421085 | ARSB | eQTL | ARSB |
|  |  |  | mRNA | ARSB |
| 39 | rs10858282 | COL5A1 | mRNA | COL5A1 |
| 40 | rs4766453 | CUX2 | eQTL | SH2B3 |
|  |  |  | mRNA | CUX2 |
| 41 | rs9889953 | SMARCE1 | Enhancer | CASC3 |
|  |  |  | Enhancer | RARA |
|  |  |  | Enhancer | CCR7 |
|  |  |  | Enhancer | WIPF2 |
|  |  |  | Enhancer | KRTAP3-2 |
|  |  |  | eQTL | CCR7 |
|  |  |  | eQTL | SMARCE1 |
| 42 | rs1256341 | ALPL | mRNA | ALPL |
| 43 | rs6583823 | KIF11 | eQTL | HHEX |
|  |  |  | mRNA | IDE |
|  |  |  | mRNA | KIF11 |
| 44 | rs3824735 | KIF11 | eQTL | HHEX |
|  |  |  | mRNA | IDE |
|  |  |  | mRNA | KIF11 |
| 45 | rs6602820 | PRKCQ | mRNA | PRKCQ |
| 46 | rs7255742 | CEACAM21 | Enhancer | TGFB1 |
|  |  |  | Enhancer | CEACAM21 |
|  |  |  | Enhancer | ARHGEF1 |
|  |  |  | Enhancer | CCDC97 |
|  |  |  | Enhancer | B9D2 |
|  |  |  | eQTL | BCKDHA |
|  |  |  | eQTL | CEACAM21 |
|  |  |  | mRNA | CEACAM21 |
| 47 | rs10754328 | CD2 | Enhancer | ATP1A1 |
|  |  |  | Enhancer | CD2 |
|  |  |  | Enhancer | TTF2 |
| 48 | rs10932017 | CD28 | eQTL | CD28 |
|  |  |  | Promoter | CD28 |
| 49 | rs2834679 | RUNX1 | mRNA | RUNX1 |
| 50 | rs2280234 | STAT1 | eQTL | GLS |
|  |  |  | mRNA | STAT1 |
| 51 | rs11894425 | STAT1 | eQTL | GLS |
|  |  |  | eQTL | STAT1 |
|  |  |  | mRNA | STAT1 |
| 52 | rs2066804 | STAT1 | eQTL | GLS |
|  |  |  | eQTL | STAT1 |
|  |  |  | mRNA | GLS |
|  |  |  | mRNA | STAT1 |
| 53 | rs3771300 | STAT1 | eQTL | GLS |
|  |  |  | mRNA | GLS |
|  |  |  | mRNA | STAT1 |
| 54 | rs12629751 | PPARG | Enhancer | PPARG |
|  |  |  | eQTL | PPARG |
|  |  |  | eQTL | TSEN2 |
|  |  |  | eQTL | SYN2 |
|  |  |  | mRNA | PPARG |
| 55 | rs7615916 | PPARG | Enhancer | PPARG |
|  |  |  | eQTL | PPARG |
|  |  |  | eQTL | TSEN2 |
|  |  |  | eQTL | SYN2 |
|  |  |  | mRNA | PPARG |
| 56 | rs5748470 | TXNRD2 | eQTL | TBX1 |
|  |  |  | eQTL | GNB1L |
|  |  |  | eQTL | TXNRD2 |
|  |  |  | mRNA | TXNRD2 |
| 57 | rs16930094 | ELF5 | eQTL | CAT |
|  |  |  | eQTL | GLYAT |
|  |  |  | mRNA | ELF5 |
| 58 | rs12596236 | RP11-343H19.2 | eQTL | MT2A |
|  |  |  | eQTL | MT1E |
|  |  |  | eQTL | MT1F |
|  |  |  | eQTL | MT1G |
|  |  |  | eQTL | MT1X |
|  |  |  | eQTL | HERPUD1 |
|  |  |  | eQTL | MT1M |
|  |  |  | eQTL | BBS2 |
| 59 | rs478829 | CLEC12A | eQTL | CLEC12A |
|  |  |  | eQTL | CLEC7A |
|  |  |  | mRNA | CLEC12A |
|  |  |  | Promoter | CLEC12A |
| 60 | rs10828375 | ARMC3 | eQTL | COL5A2 |
| 61 | rs2229495 | COL5A2 | mRNA | COL5A2 |
| 62 | rs1996913 | ANKS1A | eQTL | UHRF1BP1 |
|  |  |  | eQTL | ANKS1A |
|  |  |  | mRNA | ANKS1A |
| 63 | rs4475472 | YTHDF3 | eQTL | GGH |
| 64 | rs4739053 | YTHDF3 | eQTL | GGH |
| 65 | rs9358028 | CD83 | Enhancer | CD83 |
|  |  |  | Enhancer | RANBP9 |
|  |  |  | eQTL | CD83 |
| 66 | rs12677559 | PTK2 | eQTL | PTK2 |
|  |  |  | mRNA | PTK2 |
|  |  |  | Promoter | PTK2 |
| 67 | rs17722057 | RYR1 | mRNA | RYR1 |
| 68 | rs4920110 | RSPH1 | Enhancer | ABCG1 |
|  |  |  | Enhancer | UBASH3A |
|  |  |  | Enhancer | ZNF295 |
|  |  |  | eQTL | RSPH1 |
|  |  |  | mRNA | RSPH1 |
| 69 | rs7595037 | PLEK | eQTL | PLEK |
|  |  |  | eQTL | PPP3R1 |
|  |  |  | eQTL | CNRIP1 |
| 70 | rs6723149 | AC015969.3 | eQTL | PLEK |
|  |  |  | eQTL | PPP3R1 |
|  |  |  | mRNA | PLEK |
| 71 | rs1558722 | TBC1D19 | eQTL | RBPJ |
|  |  |  | mRNA | TBC1D19 |
| 72 | rs6818128 | RBPJ | eQTL | RBPJ |
| 73 | rs7671350 | RBPJ | Enhancer | RBPJ |
| 74 | rs8109559 | INSR | mRNA | INSR |
| 75 | rs10169393 | RBPJ | mRNA | AFF3 |
| 76 | rs10185087 | AFF3 | mRNA | AFF3 |
| 77 | rs897477 | NHEJ1 | eQTL | STK16 |
|  |  |  | eQTL | CYP27A1 |
|  |  |  | eQTL | ZFAND2B |
|  |  |  | mRNA | SLC23A3 |
|  |  |  | mRNA | CCDC108 |
|  |  |  | mRNA | NHEJ1 |
|  |  |  | Promoter | CCDC108 |
|  |  |  | Promoter | NHEJ1 |
| 78 | rs375288 | IRF8 | eQTL | IRF8 |
| 79 | rs245478 | CTD-2260A17.2 | eQTL | CD3EAP |
|  |  |  | eQTL | CAST |
|  |  |  | eQTL | ERAP1 |
|  |  |  | eQTL | LNPEP |
|  |  |  | mRNA | ERAP1 |
|  |  |  | Promoter | ERAP1 |
| 80 | rs2040623 | AHR | mRNA | AHR |
| 81 | rs9639288 | KCCAT333 | mRNA | AHR |
| 82 | rs6597642 | ABL1 | mRNA | ABL1 |
| 83 | rs7604115 | UGT1A5 | mRNA | UGT1A9 |
|  |  |  | mRNA | UGT1A6 |
|  |  |  | mRNA | UGT1A1 |
|  |  |  | mRNA | UGT1A4 |
|  |  |  | mRNA | UGT1A3 |
|  |  |  | mRNA | UGT1A7 |
|  |  |  | mRNA | UGT1A10 |
|  |  |  | mRNA | UGT1A8 |
|  |  |  | Promoter | UGT1A1 |
|  |  |  | Promoter | DNAJB3 |
| 84 | rs7901986 | ATF3 | Enhancer | DDIT4 |
|  |  |  | Enhancer | DNAJB12 |
|  |  |  | eQTL | CBARA1 |
|  |  |  | eQTL | DDIT4 |
|  |  |  | eQTL | DNAJB12 |
|  |  |  | mRNA | CBARA1 |
|  |  |  | Promoter | CBARA1 |
| 85 | rs12777437 | DMRT2/Pax-4 | eQTL | CBARA1 |
|  |  |  | eQTL | DDIT4 |
|  |  |  | eQTL | DNAJB12 |
|  |  |  | mRNA | CBARA1 |

Table S4. Sensitivity, specificity, and positive predictive value of the final models

|  | | sensitivity | | specificity | | positive  predictive value | |
| --- | --- | --- | --- | --- | --- | --- | --- |
|  |  | Mean | SD | Mean | SD | Mean | SD |
| SNPs Only | Post-GWAS | 0.5924 | 0.1369 | 0.6106 | 0.1463 | 0.6117 | 0.1099 |
|  | GWAS | 0.3227 | 0.1182 | 0.3985 | 0.0884 | 0.3415 | 0.1062 |
|  | SPOT | 0.5076 | 0.1304 | 0.4826 | 0.1448 | 0.4959 | 0.0784 |
| SNPs+Clinical | Post-GWAS | 0.7644 | 0.1146 | 0.7318 | 0.1152 | 0.7445 | 0.1096 |
|  | GWAS | 0.6364 | 0.1283 | 0.6614 | 0.1358 | 0.6591 | 0.1183 |
|  | SPOT | 0.7288 | 0.1347 | 0.7038 | 0.1165 | 0.7176 | 0.0765 |
