# Supplementary material for: Biological function integrated prediction of severe radiographic progression in rheumatoid arthritis: a nested case control study
Source: Arthritis Res Ther. 2017 Oct 25;19:244. doi: 10.1186/s13075-017-1414-x (PMC5655942; doi:10.1186/s13075-017-1414-x)
Supplement: Supplementary file 2 — The weighted sum of probabilities in each model. Figure S2. Receiver operating characteristic (ROC) curve for the result of replication of the final SNPs in the NARAC cohort. (PPTX 207 kb) [file 13075_2017_1414_MOESM2_ESM.pptx]

## Slide 1
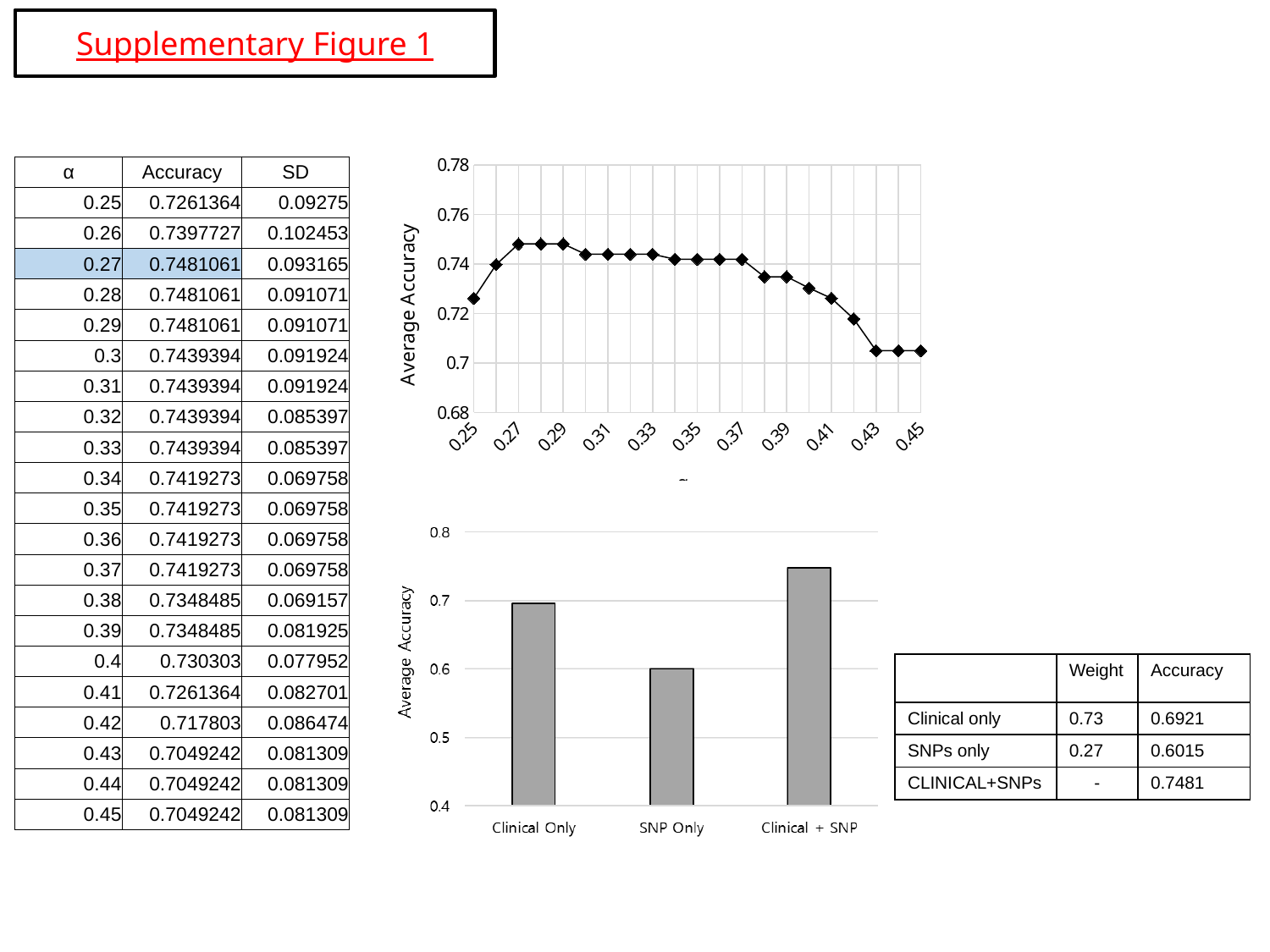

Supplementary Figure 1
### Chart
| Category | Accuracy |
|---|---|
| 0.25 | 0.72613636 |
| 0.26 | 0.7397727 |
| 0.27 | 0.74810606 |
| 0.28000000000000003 | 0.74810606 |
| 0.28999999999999998 | 0.74810606 |
| 0.3 | 0.74393939 |
| 0.31 | 0.74393939 |
| 0.32 | 0.74393939 |
| 0.33 | 0.74393939 |
| 0.34 | 0.74192727 |
| 0.35 | 0.74192727 |
| 0.36 | 0.74192727 |
| 0.37 | 0.74192727 |
| 0.38 | 0.73484848 |
| 0.39 | 0.73484848 |
| 0.4 | 0.73030303 |
| 0.41 | 0.72613636 |
| 0.42 | 0.71780303 |
| 0.43 | 0.70492424 |
| 0.44 | 0.70492424 |
| 0.45 | 0.70492424 || α | Accuracy | SD |
| --- | --- | --- |
| 0.25 | 0.7261364 | 0.09275 |
| 0.26 | 0.7397727 | 0.102453 |
| 0.27 | 0.7481061 | 0.093165 |
| 0.28 | 0.7481061 | 0.091071 |
| 0.29 | 0.7481061 | 0.091071 |
| 0.3 | 0.7439394 | 0.091924 |
| 0.31 | 0.7439394 | 0.091924 |
| 0.32 | 0.7439394 | 0.085397 |
| 0.33 | 0.7439394 | 0.085397 |
| 0.34 | 0.7419273 | 0.069758 |
| 0.35 | 0.7419273 | 0.069758 |
| 0.36 | 0.7419273 | 0.069758 |
| 0.37 | 0.7419273 | 0.069758 |
| 0.38 | 0.7348485 | 0.069157 |
| 0.39 | 0.7348485 | 0.081925 |
| 0.4 | 0.730303 | 0.077952 |
| 0.41 | 0.7261364 | 0.082701 |
| 0.42 | 0.717803 | 0.086474 |
| 0.43 | 0.7049242 | 0.081309 |
| 0.44 | 0.7049242 | 0.081309 |
| 0.45 | 0.7049242 | 0.081309 |
| | Weight | Accuracy |
| --- | --- | --- |
| Clinical only | 0.73 | 0.6921 |
| SNPs only | 0.27 | 0.6015 |
| CLINICAL+SNPs | - | 0.7481 |

## Slide 2
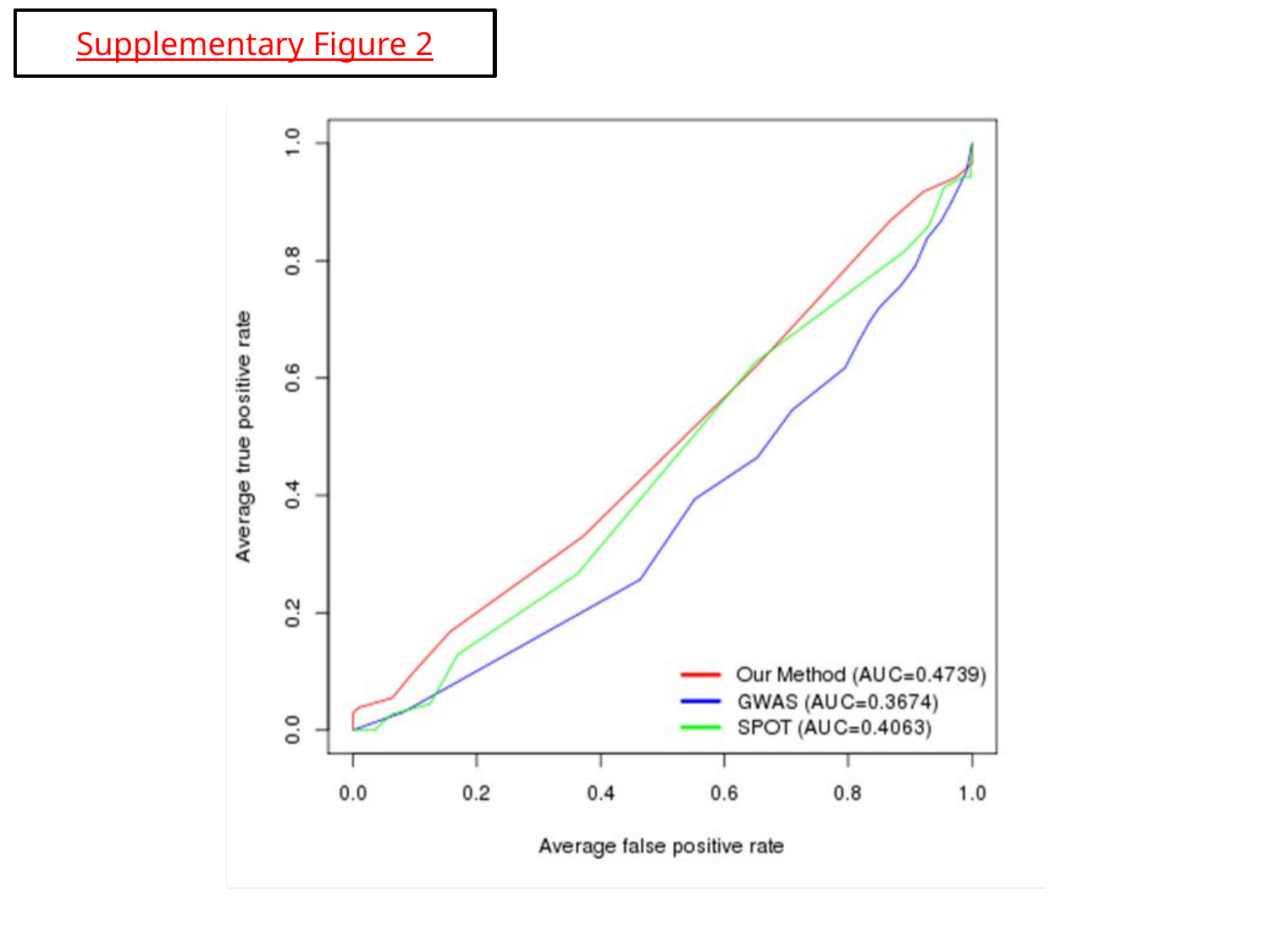

Supplementary Figure 2
